# Risks and perception of non-communicable diseases and health promotion behavior of middle-aged female immigrants in Japan: a qualitative exploratory study

**DOI:** 10.1186/s12905-020-00955-1

**Published:** 2020-05-01

**Authors:** Yasuko Nagamatsu, Edward Barroga, Yumi Sakyo, Yukari Igarashi, Yuko Hirano O

**Affiliations:** 1 Graduate School of Nursing Science, St. Luke’s International University, 10-1 Akashi-cho, Chuo-ku, Tokyo, 104-0044 Japan; 2 Institute of Biomedical Sciences, Nagasaki University, 1-7-1 Sakamoto, Nagasaki City, Nagasaki, 8528520 Japan

**Keywords:** Immigrant, Non-communicable disease, Health promotion, Women, Breast cancer, Cervical cancer

## Abstract

**Background:**

Ensuring good health of immigrants is a serious issue across countries, including Japan. This study focused on the health of middle-aged female immigrants in Japan who experienced changes to their health as well as an increased risk of non-communicable diseases. Specifically, the study aimed to clarify the risks and perceptions of non-communicable diseases and health promotion behavior of middle-aged female immigrants in Japan.

**Methods:**

This investigation used an exploratory design. The participants were a purposive sample of 35 middle-aged female immigrants (age ≥ 40 years) living in urban and rural areas of Japan. Data were generated using mixed methods. A quantitative approach provided data of their risks of non-communicable diseases. Focus group discussions provided insights to identify their health promotion perceptions.

**Results:**

Blood pressure measurement revealed that 29% of the immigrants had hypertension, 29% had a body mass index of > 30, and 71% had an abdominal girth of > 80 cm. About 31% had a history of chronic disease and 34% had regular medication. There were 80% who received regular health check-up, 49% who received breast cancer screening, and 34% who received cervical cancer screening. The focus group discussions indicated that the middle-aged female immigrants recognized the threat of non-communicable diseases. However, they lacked knowledge about the prevention of non-communicable diseases, and they felt that non-communicable diseases were unavoidable. They also failed to understand the benefits of health promotion behavior. The study revealed that the monolingual Japanese health service prevented immigrant women from understanding their health check-up and cancer screening results, and how to utilize the health service system.

**Conclusions:**

Middle-aged female immigrants in Japan had potential risks of non-communicable diseases, and recognized their threat. These settled immigrant women received health check-ups and cancer screenings with the support of their family, and consequently attained the same level of adherence as that of Japanese women. However, lack of knowledge about health promotion and its benefits and the absence of a culturally sensitive health service system for immigrants in Japan constrained their health-promotion behavior. Sociocultural multilingual-tailored interventions including interpretation services by care providers with cultural sensitivities must be developed.

## Background

Ensuring good health of migrants is a serious global issue. This is well recognized by the World Health Organization (WHO) which advocated the health promotion of migrants at its 61st World Health Assembly [1]. Access to health care is crucial for ensuring migrants’ good health. Unfortunately, illegal status [2], language problems [3], cultural differences [3, 4], lack of basic information [5], inadequate health literacy, [6] and financial difficulty [7, 8] act as barriers to health care access.

There are currently 2,829,416 migrants in Japan, and this number continues to grow [9]. As of 2019, the number of migrants who became permanent residents, referred to as immigrants, in Japan was about 1,160,000 and 64% were women over the age of 40 years [9]. Although immigrants are a diverse group [9], Japan is not a culture that has emerged from an influx of various cultures, as contrasted with Australia, Canada or the United States. Quite the contrary, Japan has a past history of closed borders and tight control of in-migration. Furthermore, the language and customs are thought to be unique and a defining part of the Japanese national identity. This often poses a challenge to immigrants who are attempting to become part of the culture [10, 11]. Many immigrants in Japan reported experiencing similar difficulties as migrants because of the different culture or language and the unfamiliar health services that involve a complicated Japanese insurance system [12–15]. Even long-term immigrants had difficulties in communicating with health providers [16].

Although Japan has a proactive response to its aging population [17], the cultural and language differences may place the middle-age female immigrant population at a disadvantage when facing issues of aging. Middle-aged women have various physiological, physical, cognitive and social changes associated with aging that may increase their risk of non-communicable diseases (NCDs) [18–20]. One study reported that there was a risk of obesity in relation to NCDs among Filipina migrants in Japan [21]. Health promotion strategies with a strong focus on disease prevention are needed to empower women to reduce the risks of NCDs [22]. However, there has been little research regarding the risks of NCDs of middle-aged female immigrants in Japan as well as their perception of NCDs and health promotion behaviors. Thus, a major issue that remains to be addressed in Japan is how to promote the health of migrant women. This study aimed to clarify the risks of and perceptions about NCDs as well as the health promotion behavior of middle-aged female immigrants in Japan.

### Definition

In this study, the term immigrants refer to persons who were born in a country other than Japan, migrated to Japan, and granted a permanent resident status.

### Theoretical framework

The theoretical framework guiding this study was the health belief model because it predicts people’s health-related behavior to prevent, screen for, and control illness conditions [23]. The key constructs of the health belief model are perceived susceptibility, perceived severity, perceived benefits, perceived barriers, cue to action and self-efficacy. Diverse demographic, socio-psychological, and structural variables may influence perceptions and indirectly influence health-related behavior.

## Methods

### Study design

We used an exploratory mixed-method design consisting of a quantitative approach to clarify the risks of NCDs and a qualitative approach to identify the perceptions NCDs and health promotion behaviors of middle-aged female immigrant in Japan. There were no previous studies on the perspectives of middle-aged female immigrant regarding their risks and perceptions of NCDs and health promotion behavior. For that reason we used focus group discussions (FGDs), which are suitable for obtaining a wide range of opinions and are therefore helpful tools in assessing group needs. FGDs work particularly well to explore perceptions, feeling, and thinking about issues, ideas, products, services or opportunity [24]. Also, focus groups enable people to ponder, reflect, and listen to experiences and opinions of others...this interaction helps participant’s compare their own personal realities to those of others [24].

### Study setting

This study was conducted in an urban area (i.e., Chiba prefecture) and a rural area (i.e., Yamagata prefecture) in Japan. For the urban setting, we recruited participants from a church with about 200 immigrants in Chiba which has 200,000 immigrants. We distributed the invitation to 41 eligible immigrant women after church mass. For the rural setting, we conducted the study with an immigrant group in Yamagata prefecture, which has 6000 immigrants. We distributed the invitation to 15 eligible female members. A total of 35 immigrant women agreed to participate in this study.

### Participants

We invited a purposive sample of immigrant women living in Japan who were 40 years or older who had attained the legal status of permanent resident either though marriage or their employment. Excluded were women who were unable to communicate in either Japanese or English.

### Data collection

Data were collected from October 2016 to November 2016. The data consisted of a questionnaire survey, physical measurements, and FGDs. For the quantitative data, participants completed a self-administered questionnaire documenting their country of origin, age, marital status, years of being an immigrant, experience of having a general check-up, screening for breast cancer and cervical cancer within the year, smoking and drinking history, history of chronic disease and regular medication, and intention of joining a health promotion program. Participants’ height, weight, abdominal girth, and blood pressure were also measured. Body Mass Index (BMI) was calculated using weight and height. Based on the WHO standard [25], a person with a BMI of ≥30 or an abdominal girth of ≥380 cm was considered obese. A person with a blood pressure of > 140 mmHg over 90 mmHg was identified as having hypertension.

For the focus groups, Krueger [24] suggested conducting 3 to 4 group discussions with 5 to 8 participants. A Japanese-English bilingual researcher (YN) facilitated the discussions. The FGDs were conducted in English and Japanese. Two Japanese research assistants obtained verbal consents to take notes and audiotape the interviews. As Table 1 shows, the facilitators used the following questions to guide the semistructured interview [26]: (a) Please described the community in which you live; (b) What are your perceptions of NCDs?; (c) How do you prepare for or prevent NCDs? Various probing questions were used to elicit clarification or expansion of the participants’ responses.

**Table 1:** Focus group discussion questions

a. Please described the community in which you live	
b. What are your perceptions of NCDs?	
c. How do you prepare for or prevent NCDs?	

The first question was general and was used as an ice breaker to stimulate further discussion and put the participants at ease while encouraging them to interact normally with the facilitator. The FGDs lasted for approximately 50–60 min. The facilitator transcribed the interviews verbatim. After conducting 3 FGDs with 7 or 8 participants in each group, the full range of ideas was collected and no new information was forthcoming as we had reached data saturation and thus concluded the FGD.

### Data analysis

Descriptive data of the demographic and biomarkers included percents, totals and ranges and were analyzed using SPSS 24. BMI was calculated using the USA Center for Disease Control Adult BMI online calculator. FGD data were analyzed objectively and systematically according to the steps of analysis described by Krueger [24]. We used the classic analysis strategy to identify themes and categorize results [24]. The following steps were taken to analyze the focus group data: (1) printed the transcripts and noted the number of participants who iterated each quote; (2) read each quote and collected the quotes for which [a] participants answered the question the interviewer asked, or [b] said something important about the topic; (3) provided explanatory encoding for every sentence or paragraph related to the risks of NCDs or health promotion needs so that similar sentences or paragraphs shared the same code; (4) generated categories and subcategories based on the similarity of each code; (5) discussed the process for generating categories among the authors who were experienced qualitative researchers (YN, YS, YI, YH) to enhance the trustworthiness of data analysis; and (6) revised the naming or classification of categories based on the discussion. Although we had only minor disagreements between authors, we went back to the original quotes and discussed the coding until agreement was reached. The credibility of the results was ensured by triangulating different sources of information, reviewing disconfirming evidence, researcher flexibility, collaborating with participants, and auditing by the academic advisor [27].

### Ethical considerations

This study observed all standards for protection of human subjects as set forth by the Declaration of Helsinki. The heads of the local women’s immigrant groups granted permission to conduct the study. Research assistants obtained written informed consent from all the participants after they had received oral and written information about the study. The participants were also informed that they could voluntary stop their participation at any time without any questions or repercussions. Verbal consent to audiotape the interviews was obtained. Anonymity of their responses was assured. Each informant confirmed her participation in writing. Ethical approval for the study was obtained from the Research Ethics Committee of St. Luke’s International University, Japan (Approval number: 16-A034).

## Results

### Characteristics of participants

Table 2 shows the participant characteristics. All the participants were women with a mean age of 50.6 years and a mean residence duration in Japan of 19.5 years. The countries of origin were the Philippines, China, Korea, Thailand, and the United States of America. The majority of the participants were married and from the Philippines.

**Table 2:** Characteristics of the participants (*N* = 35)

Characteristics		
**Age** (years)		50.6 ± 7.6
**Duration of residence** (years)		19.5 ± 6.9
**Country of origin**	Philippines	28
	China	3
	Korea	2
	Thailand	1
	United States of America	1
**Marital status**	Single	2
	Married to Japanese	15
	Married to non-Japanese	14
	Divorced	2
	Widowed	2
**Chronic disease**	With chronic disease	11
	Without chronic disease	24
**Regular medication**	Yes	12
	No	23

### Risks of NCDs

There were 29% with hypertension, 29% with a BMI of > 30, and 71% with an abdominal girth of > 80 cm. Approximately 80% received a regular health check-up, 49% received breast cancer screening, and 34% received cervical cancer screening. About 31% had a history of chronic disease and 34% took prescribed medications.

Guided by the health belief model, the qualitative data about the perceptions of NCD risks and health promotion behavior were classified into four categories: (1) Non-communicable diseases as threat; (2) Health promotion behaviors; (3) Barriers related to health promotion behavior; (4) Accelerator of health promotion behavior. Details are shown in Table 3.

**Table 3:** Perceptions of NCD risks and health promotion behaviors to prevent NCDs

Non-communicable diseases as threat	Something causes serious consequences
	Unavoidable
	Need effort to prevent
Health promotion behaviors	Exercise
	Healthy diet
	Taking medicine prescribed by a doctor
	Sleep and rest
	Attend cancer screening
Barriers related to health promotion behavior	Benefits of health promotion behavior not understood
	Lack of knowledge about methods of health promotion behavior
	Passive attitude towards health promotion from country of origin
	Monolingual health service inappropriate
Accelerator of health promotion behavior	Suggestion for health promotion by family and friends
	Peer support
	Invitation of health promotion activity from government
	Supportive health provider

### Non-communicable diseases as threat

#### Something causes serious consequences

All of the middle-aged female immigrants recognized that NCDs could cause serious consequences because their families or friends experienced NCDs and they had learned about the danger of NCDs in Japan. Even though they were aware that NCDs were dangerous, it remained unclear why they experienced NCDs or how NCDs could be prevented.

“*I know that stroke is very dangerous. It killed my brother.* ”

*“My Japanese friends are so concerned about blood pressure, blood sugar and weight. I heard that hypertension is dangerous. I am so nervous. But how can I prevent it? Is there anything we can do to prevent it?”*

#### Unavoidable

Many immigrant women felt that NCDs were unavoidable because some NCDs were experienced in the same household and because NCDs were common in their community.

*“I am so scared of stroke. My father and brother had stroke. I am afraid that I may have it too. It is a family disease, I guess.”*

“*You will have them (NCDs) when you get old. It is natural. Many people have them in my country.*”

#### Need effort to prevent

Some immigrant women believed that NCDs could be prevented if a person tried to do so even though it was not easy.

“*We must work hard to keep ourselves healthy. We cannot eat, drink or behave like younger ones if we want keep ourselves healthy. It is hard*.”

*“My blood pressure was high. My doctor advised me to lose weight and take medicine. It was not easy to lose weight. But I accomplished it. Now I do not need a medicine for blood pressure. It is controlled.”*

### Health promotion behaviors

The participants engaged in some forms of health promotion behaviors such as exercise, healthy diet, taking medication prescribed by a doctor, sleep and rest and attending cancer screening.

#### Exercise

A group of immigrants organized an exercise class by themselves because there were no exercise classes for non-Japanese speaking residents, and they also claimed that it is easier to continue exercise if they do it with a friend.

*“I invite an English speaking instructor to my office every month and have an exercise class with my immigrant friends. It is fun if you have friends to do with.”*

#### Healthy diet

Some immigrant women tried to eat in healthy ways. Many immigrant women felt that the Japanese eating styles were healthier than the original eating styles from their countries. However, Filipino participants mentioned that eating Japanese rice made them fat.

*“Filipinos love eating. It is our culture to eat together … I rather eat vegetable and fish like Japanese do but I am trying not eat much rice. It makes me fat.”*

*“I heard that hypertension is dangerous. I am so nervous. But how can I prevent it? Is there anything we can do to prevent it?”*

#### Taking medicine prescribed by a doctor

Some immigrant women were taking the prescribed medicine for NCDs. The most common NCD was hypertension and the prescribed hypertensive medication were taken. The physician did not explain how the medication worked, so she was not very motivated to take it.

*"I am taking a medicine because my blood pressure is high. It is Ok to take when my pressure is high but I do not like to keep taking medicine regularly. But my doctor says I must take it everyday. It is hard."*

### Barriers related to health promotion behavior

#### Benefits of cancer screening or health check-ups not understood

Some participants did not know why people would benefit from cancer screening or health check-ups because receiving a healthy result of health check-up made them feel it is useless.

“*I always have “normal” as a result of my health check-up. Why do I have to go to the hospital for assurance of “normal”? It is waste of time and resource*”

One participant confessed that she did not attend cancer screening because she was scared to be diagnosed. In her country, cancer is fatal and she believed that there is no point to detect it at an earlier stage.

*“I am simply scared to be diagnosed with cancer...that is why I do not go (to screening).”*

#### Lack of knowledge about methods of health promotion

Some immigrant women did not have any idea how to promote their health because they were young and healthy when they departed from their country of origin and did not have an opportunity to learn about health promotion in Japan.

*“I know I am getting old and want to do something to keep myself healthy. But how? In my country, old people did not care about blood pressure.”*

#### Passive attitude towards health promotion from country of origin

Some immigrant women hesitated to visit the hospital because of their cultural background.

*“Japanese really love to visit the hospital. We visit the hospital only when we have a serious illness. Why do I have to visit a doctor when I have no symptom?”*

*“We believe in God. We pray rather than going to the hospital. If I get a cancer, it is my fate.”*

#### Language barriers obstructing health service

All immigrant women demanded the improvement of Japanese health information, which was only written and spoken in Japanese. Monolingualism in the Japanese health service prevented immigrant women from attending the check-up or cancer screening, or if they did they failed to understand the results of the medical examination. Participants wanted know about their health in detail.

*“I got an invitation form of cancer screening written in Japanese which I do not read. It contained a list of clinics, however, I did not know where I can see the doctor who speaks English.”*

*“To keep my health, I went to breast cancer screening. The problem was that I did not fully understand the results. I do not read Japanese. My husband said it was OK. What I wanted to know was how it was OK and what to do to keep me away from the breast cancer … .It is my health, not my husband’s.”*

### Accelerator of health promotion behavior

#### Suggestion for health promotion by family and friends

Suggestion by the participants’ family and friends strongly urging them to attend the health check-up and cancer screening motivated them to attend.

“*My husband and son asked me to [go to the] hospital for check-up. That is why I went.”*

#### Invitation of health promotion activity from government

Invitation for a free check-up or cancer screening from the government was a strong incentive for immigrant women. This was especially true when it was sent through the employer.

“*I got an invitation of health check-up. They said it was free. I was sorry to waste it...so I went*.”

#### Peer support

Immigrant women wanted to attend health promotion events with their friends who shared their language and culture.

*“I want to have friends to share the health problem and support each other. I need a community where we are comfortable and empowered.”*

#### Supportive health provider

Immigrant women felt empowered to behave in healthy ways when trusted health providers supported them.

“*I am lucky. My doctor is very nice person. He try to speak English using dictionary. Not good but I know he try to communicate with me … I feel he really try to help me. That is why I do not skip his medical appointment.”*

## Discussion

This exploratory study aimed to clarify the perceived risks of NCDs and the health promotion behaviors of middle-aged female immigrants in Japan.

### Perceptions of NCD risk and health promotion behaviors

Notably, slightly less than one-third of the female immigrant participants in this study were found to have risks of hypertension, a BMI of > 30, and an abdominal girth of > 80 cm as NCDs. Chronic disease was also found among one-third of the female immigrant participants, and some took regular medication.

Interestingly, 80% of the study participants received a health check-up in the previous year, which is higher than the 53% rate of the Japanese population who received a health check-up [28]. Moreover, 49% of the study participants received breast cancer screening, which is slightly higher than the 36.9% breast cancer screening rate of the Japanese population. Unfortunately, only 34.3% of the study participants received cervical cancer screening, which is nearly the same as the 33.7% cervical cancer screening rate of the Japanese population [29]. Interestingly these screening rates are different from those of studies in other countries, which reported a lower adherence to cancer screening by immigrant women than by non-immigrant women [30–32].

Several reasons may underlie such differences. The first reason may be sample bias. Our sample size was small and many of the participants have been in Japan for a considerable time, which implies that they have become familiar with the health service in Japan. Also, the participants belonged to certain communities such as a church or an immigrant group, which provided them support to engage in health promotion activities. The second reason is the low adherence of the Japanese population to cancer screening. Nevertheless, even though the adherence rates to breast and cervical cancer screenings of the female immigrants were similar to or even higher than those of the Japanese population, the rates are still lower than those of other developed countries [33]. The specific reasons for the low rates of adherence to cancer screening in Japan remain to be elucidated. One possible reason is that the Japanese universal health insurance system has made it possible for all residents including immigrants to have access to reasonable health service any time. Interestingly, even though immigrant women achieved the same rate of adherence to cancer screening, our study showed low satisfaction with health check-ups and cancer screenings, which eventually compounded the negative behavior of complacency in pursuing health promotion activities.

### Perception of NCDs and health promotion behaviors

As noted earlier, according to the concepts of the heath belief model, behaviors to prevent disease are related to the following 6 constructs: *perceived susceptibility*, *perceived severity*, *perceived benefits*, *perceived barrier*, *cue to action*, and *self-efficacy* [34]. In this current study, immigrant women recognized the danger of NCDs and may have feared that they could have one which indicated their recognition of susceptibility and severity of NCDs, and potentially increased their motivation for health promotion behaviors [35]. However, some immigrant women failed to understand the benefits of health promotion behaviors such as going to health check-ups or attending cancer screenings. Moreover, barriers such as lack of knowledge about the methods and language difficulties while attending the Japanese health service most likely contributed to their lack of developing effective health promotion behaviors.

The barriers described by the immigrant women were similar to the those of previous studies such as language barrier [35, 36], health care system-related barriers [35, 36], and lack of knowledge [37, 36]. Invitations for health check-ups or cancer screenings, and suggestions by family or friends were effective cues for action. Monolingualism in the Japanese health service was not only a barrier for health promotion but also one that potentially damaged the participants’ self-efficacy because immigrant women were incapable of speaking-up, asking questions, understanding health information and making decisions about their own health. According to the health belief model, for immigrant women to engage in health promotion behaviors to prevent NCDs (outcome), they must believe that health promotion behaviors will benefit their health (outcome expectation) and also that they are capable of health promotion behaviors (efficacy expectation) [35]. Furthermore, some immigrant women believed that NCDs can be prevented, which indicates “internal locus of control”. On the other hand, some immigrant women felt that NCDs are unavoidable and out of control, which indicates “external locus of control” [38].

To improve the health promotion of immigrant women, we must start by facilitating their understanding of the benefits of health promotion. The benefits of health promotion should be provided within a context that immigrant women could understand. In terms of effective interventions regarding cancer screening promotion for immigrants, previous studies [39–41] found that sociocultural-tailored interventions must be developed. Health care providers should recognize that immigrant women may have different beliefs and values [42]. Because of the deleterious effects of language and knowledge barriers, there is an urgent need to develop multi-language health services in Japan. Interpreters with correct technique and in the right environment to facilitate high-quality communication based on a trustful relationship ensuring confidentiality are needed [43]. Furthermore, community participative communication interventions are recommended for vulnerable populations such as immigrants [44].

### Limitations

This exploratory study assessed only 35 female immigrant women from one urban and one rural area of Japan. As the number of the participants was small and the majority of women were Filipino, responses were likely influenced by that culture. Although focus groups provide rich data, they can also make it more difficult for some participants to voice dissenting opinions. In this study, every attempt was made to support all viewpoints. Moreover, immigrant women who are from other countries and who do not speak either English or Japanese may have different health promotion needs. Despite the small sample size and the majority of the participants coming from the Philippines, our findings have some transferability given the similarities of findings from other studies, particularly language barriers within the health care system. Furthermore, because little was known about the risk and perception of NCDs of female immigrants in Japan, the explorative study design was chosen. A study using focus group discussion is not intended to generalize [24] .Although the health promotion needs of the participants in this study may not accurately reflect those of all middle-aged immigrant women in Japan, these reported needs provide a good background for identifying further relevant research areas.

## Conclusions

This study clarified that the participating middle-aged female immigrants in Japan have potential risks of NCDs, and that they have recognized the threat of NCDs. The settled immigrant women received health check-ups and cancer screenings with the support of their family, and consequently attained the same level of adherence as that of Japanese women. However, some of the participants failed to understand the benefits of health promotion. Moreover, the culturally insensitive health service system for immigrants in Japan constrained their health promotion behaviors. Despite the need for additional research, it is recommended that sociocultural multilingual-tailored interventions including interpretation services by care providers with cultural sensitivities must be developed and integrated urgently.

## Data Availability

The datasets used and analyzed in the current study are available from the corresponding author upon reasonable request.

## Abbreviations

NCDs: Non-Communicable Diseases
WHO: World Health Organization
BMI: Body Mass Index
